# Sociodemographic and Clinical Profile of Poisoning Cases in a Tertiary Care Hospital: A Retrospective Study With Psychiatric Correlates

**DOI:** 10.7759/cureus.101946

**Published:** 2026-01-20

**Authors:** Sri Ridanya S, Sabari Sridhar OT, Chamelee Anbu, Nishfa Saleem, Aiswarya M Nair

**Affiliations:** 1 Psychiatry, Sri Ramachandra Institute of Higher Education and Research, Chennai, IND; 2 Internal Medicine, BloomLife Hospital, Chennai, IND

**Keywords:** depressive disorder, emergency medicine, epidemiology, good health and well-being, mental health, poisoning, psychiatric comorbidity, retrospective study, self-harm, tertiary care hospital

## Abstract

Background and objective

Poisoning remains a significant public health concern in underdeveloped countries, particularly among young adults. Intentional poisoning is increasingly recognized as a manifestation of psychological distress and difficulty coping with daily stressors; however, data on the psychiatric aspects of poisoning are limited. This study aimed primarily to identify psychiatric comorbidities and psychosocial stressors associated with poisoning. Secondary objectives included describing the sociodemographic characteristics, clinical profile, types of poisoning, and clinical outcomes of affected patients admitted to a tertiary care hospital.

Methodology

This retrospective study was conducted at a tertiary care hospital in Chennai. Medical records of patients admitted with poisoning between January 2018 and January 2023 were reviewed. Of 381 records retrieved, 36 were excluded due to incomplete data, resulting in a final sample of 345 patients. Adults aged ≥18 years with a diagnosis of poisoning based on the ICD-10 coding were included. Exclusion criteria were age <18 years, pregnancy, food poisoning, animal bites, chronic toxicity, and patients declared dead on arrival. Data on sociodemographic variables, type of poisoning, psychosocial stressors, psychiatric diagnoses, and clinical outcomes were extracted from electronic medical records. Psychiatric diagnoses were obtained from consultation-liaison psychiatry notes documented during hospitalization and made by qualified psychiatrists using ICD-10 criteria following clinical assessment. Data were collected using a structured abstraction tool and analyzed using IBM SPSS Statistics for Windows, Version 22.0 (Released 2013; IBM Corp., Armonk, NY, USA). Normality was assessed using the Shapiro-Wilk test. Independent sample t-tests and chi-square tests were used for continuous and categorical variables, respectively, and Pearson’s correlation was applied to assess associations between psychiatric diagnoses and selected clinical variables.

Results

Most patients were aged 18-30 years (47.8%, n = 165), female (70.1%, n = 242), married (71.3%, n = 246), and from socioeconomic class II (53%, n = 183). The most common poisoning agents were corrosives (22.9%, n = 79) and benzodiazepines (19.1%, n = 66). Poisoning was planned in 95.9% (n = 331) of cases and occurred at home in 96.5% (n = 333). Psychiatric morbidity was identified in 42.6% (n =147) of patients, most commonly mood disorders (25.2%, n = 88), with depressive disorder accounting for 21.7%. Adjustment and stress-related disorders (3.8%) and personality disorders (3.8%) were also noted. Age group (p = 0.002) and presence of psychosocial stressors (p = 0.041) showed significant associations with psychiatric diagnoses.

Conclusion

Intentional poisoning was more frequently observed among young women, often in the context of interpersonal, familial, and marital stressors. A substantial proportion of patients had underlying psychiatric morbidity, predominantly mood disorders. These findings underscore the importance of integrated psychiatric evaluation and early psychosocial intervention in the management and prevention of poisoning, particularly among vulnerable populations.

## Introduction

Poisoning is one of the significant causes of morbidity and mortality worldwide, with underdeveloped and developing countries bearing a high burden. It is a major public health concern that affects people of all ages, although it is more common in young age [1]. A Nepalese study evaluating psychiatric manifestations in patients admitted following intentional self-harm observed that 30 out of 73 (41.1%) had depression spectrum disorder (including depression, dysthymia, and adjustment disorder) and 19 out of 73 (26%) had a personality disorder [2]. In India, poisoning is not just a medical emergency, but it is also a complicated societal problem that is affected by many factors, such as socioeconomic position, work exposure, psychological stressors, and availability of poisonous chemicals [3]. According to the World Health Organization (WHO), poisoning kills hundreds of thousands of people every year [4]. In South and Southeast Asia, one of the main causes of poisoning is intentional. In India, this problem is worsened by easy access to agricultural chemicals, drugs, and home corrosives [5]. This has led to a broad range of poisoning cases coming to emergency rooms [6].

The cause and type of poisoning vary by geography, the availability of the compounds, and the demography of the people that live there [7]. In rural regions, pesticides and insecticides are more widespread. In urban areas, however, pharmaceutical overdoses are becoming more common [8]. A lot of instances include intentional poisoning, which is generally linked to mental health issues and emotional dysregulation [9]. It is harder to deal with poisoning when there are underlying psychological conditions that are not detected or addressed. People who intentionally poison themselves often have depression, anxiety, personality disorders, or drug use disorders, which would require a collaborative team involving specialists from varied fields for both diagnosis and treatment [10,11].

Doctors in India are realizing that mental status examination should be a part of routine assessment for those who are being admitted for poisoning, as there is not much information available on how common and what kind of mental illnesses are in this group, especially from tertiary care facilities. Finding psychosocial stresses and mental illnesses early on may assist these individuals to have better long-term results and lower the chances of recurrence in their attempts [12-14].

The primary objective of this study was to identify psychiatric comorbidities and psychosocial stressors associated with poisoning. Secondary objectives included describing the sociodemographic characteristics, clinical profile, types of poisoning, and clinical outcomes of patients admitted to a tertiary care hospital. Although there are several studies on trends of poisoning, only a few have focused specifically on psychiatric morbidity and psychosocial stressors. This research aims to add to the gap in the regional data by looking at the trends and mental health patterns of these individuals.

## Materials and methods

This retrospective study was conducted at Sri Ramachandra Institute of Higher Education and Research (SRIHER), Chennai, a tertiary care multispecialty teaching hospital with approximately 1,800 beds. The institution serves a wide catchment area, comprising urban, semi-urban, and rural populations from Chennai and the surrounding districts of Tamil Nadu. The emergency department manages a high volume of acute medical and toxicological cases, including poisoning, providing a suitable setting for this study. It looked at records of patients who were admitted between January 2018 and January 2023 (inclusive) for poisoning. The primary objective of this study was to identify psychiatric comorbidities and psychosocial stressors associated with poisoning. Secondary objectives included describing the sociodemographic characteristics, clinical profile, types of poisoning, and clinical outcomes of patients admitted to a tertiary care hospital. The study was conducted and reported in accordance with the Strengthening the Reporting of Observational Studies in Epidemiology (STROBE) guidelines.

The research included all adult patients who were 18 years old and above and had a documented diagnosis of poisoning when they were hospitalized. We carefully reviewed the medical records, utilizing data stored in the computerized hospital information system, and adhered to the ICD-10 coding criteria. Patients who were under 18 years old, pregnant, had food poisoning, animal bites, long-term medication or chemical toxicity, or were pronounced dead on arrival to the hospital were not included in the research.

The data on sociodemographic profile, including name, age, sex, marital status, job, religion, and living arrangement (alone, with family, in a hostel, or with friends), were collected. Socioeconomic status was classified using the Modified Kuppuswamy Scale, which categorizes individuals based on education, occupation, and monthly income, with class II representing an upper-middle socioeconomic status [15]. The clinical variables included the type of poisoning (corrosives, insecticides, pharmaceuticals, sedatives, analgesics, etc.), the route of exposure (oral or inhalational), and the treatment outcome.

The study also looked at psychosocial stressors or triggers that led to poisoning (interpersonal, family, financial, work-related, etc.), and the location of the poisoning (home, hostel, workplace, etc.). We also collected information on the average length of stay in the hospital and the patient's treatment outcome (recovery or discharge against medical advice). Psychiatric comorbidities were identified from consultation-liaison psychiatry records (i.e., documentation of psychiatric assessments conducted for patients admitted under non-psychiatric departments), and any psychiatric diagnosis established during hospitalization were retrieved from electronic medical documentation. These diagnoses were made by qualified psychiatrists based on ICD-10 diagnostic criteria following clinical psychiatric assessment. We also looked at the details of earlier suicide attempts, how long it took between attempts, and the reason given for the attempts.

Data were analyzed using IBM SPSS Statistics for Windows, Version 22.0 (Released 2013; IBM Corp., Armonk, NY, USA). Data normality was assessed using the Shapiro-Wilk test. While some continuous variables showed a non-normal distribution, parametric tests (Independent samples t-test and Pearson’s correlation) were employed because the study’s large sample size (N = 345) satisfies the Central Limit Theorem. According to this theorem, the sampling distribution of the mean approaches normality as the sample size increases (typically n > 30), making parametric tests robust against non-normality in large cohorts. Two-tailed independent samples t-tests were applied to compare time to hospital presentation (hours) and duration of hospital stay (days) between patients with and without psychiatric diagnoses. Chi-square tests were used to assess associations between psychiatric diagnosis status and categorical variables, including sex, age group, and type of psychosocial stressor. Pearson’s correlation analysis was performed to evaluate the association between psychiatric diagnosis status and the interval between poisoning attempts, as well as selected medical comorbidities. A p-value < 0.05 was considered statistically significant. The Institutional Ethics Committee at Sri Ramachandra Institute of Higher Education and Research (SRIHER) gave the study its approval (Reference No.: IEC -N1/23/AUG/89/93). The approval was valid for the period covering the conduct of the retrospective study.

## Results

During the five-year study period, 381 cases of poisoning were reviewed, of which 36 were excluded due to incomplete data. Table 1 presents the sociodemographic characteristics of the remaining 345 patients. Nearly half of the participants were aged 18-30 years (47.8%, n = 165), and women constituted 70.1% (n = 242) of the study population. Following treatment, 82.6% (n = 285) of patients recovered. Housewives formed the largest occupational group (42%, n = 145). Most patients were married (71.3%, n = 246) and belonged to socioeconomic class II (53%, n = 183). The majority lived with their families (95.7%, n = 330), and Hinduism was the most common religion (94.5%, n = 326).

**Table 1:** Sociodemographic profile of patients with poisoning at a tertiary care hospital in Chennai (N = 345) *Occupations documented in medical records without further role specification.

Variable	Category	Frequency	Percentage (%)
Age (years)	18-30	165	47.8
	31-45	77	22.3
	46-60	61	17.7
	61-75	36	10.4
	76-90	6	1.7
Gender	Female	242	70.1
	Male	103	29.9
Treatment outcome	Recovered	285	82.6
	Discharged against medical advice	60	17.4
Occupation	Housewife	145	42
	Student	81	23.5
	Driver	27	7.8
	Retired	19	5.5
	Information technology (IT) professional	12	3.5
	Business	10	2.9
	Nurse	11	3.2
	Manager	9	2.6
	Farmer	6	1.7
	Assistant* (unspecified)	3	0.9
	Teacher	3	0.9
	Housemaid	2	0.6
	Human resources (HR)	2	0.6
	Unemployed	2	0.6
	Other	10	2.9
Marital status	Married	246	71.3
	Unmarried	94	27.2
	Widow	3	0.9
	Divorced	2	0.6
Socioeconomic status	Class II	183	53
	Class III	161	46.7
	Class I	1	0.3
Living arrangement	With family	330	95.7
	Hostel	8	2.3
	Alone	6	1.7
	With friends	1	0.3
Religion	Hindu	326	94.5
	Christian	11	3.2
	Muslim	8	2.3

Table 2 summarizes the clinical characteristics and patterns of poisoning among admitted patients. The highest number of cases occurred in May (11%, n = 38). Corrosive ingestion was the most common poison (22.9%, n = 79), followed by benzodiazepines (19.1%, n = 66), antidepressants (9.3%, n = 32), and antihypertensives (6.7%, n = 23). Most patients (78.6%, n = 271) ingested a single substance, while concurrent use of multiple substances was less frequent, with benzodiazepines (8.4%, n = 29) and alcohol (5.2%, n = 18) being the most common combinations. Interpersonal stressors were the leading triggers (40.6%, n = 140), followed by family problems (19.1%, n = 66) and marital issues (18%, n = 62); in 10.4% of cases (n = 36), no clear stressor was identified. Nearly all poisonings occurred at home (96.5%, n = 333), with oral ingestion being the primary route (98.8%, n = 341). The majority of cases (95.9%, n = 331) were intentional.

**Table 2:** Clinical characteristics of patients with poisoning at a tertiary care hospital in Chennai (N = 345) *Simultaneous intake of the primary poisoning agent with additional substances, as recorded in the medical case records.

Variable	Category	Frequency	Percentage (%)
Month of admission	May	38	11
	April	36	10.4
	July	35	10.1
	March	35	10.1
	August	33	9.6
	February	33	9.6
	September	30	8.7
	December	25	7.2
	January	25	7.2
	June	23	6.7
	October	19	5.5
	November	13	3.8
Type of poison	Corrosive	79	22.9
	Benzodiazepine	66	19.1
	Antidepressant	32	9.3
	Antihypertensive	23	6.7
	Rodenticide	22	6.4
	Paracetamol	20	5.8
	Antibiotic	13	3.8
	Nonsteroidal anti-inflammatory drug (NSAID)	11	3.2
	Unknown tablet	11	3.2
	Antipsychotic	9	2.6
	Thyronorm	9	2.6
	Antiepileptic	5	1.4
	Insecticide	5	1.4
	Mood stabilizer	5	1.4
	Other	30	8.7
Co-ingestion*	None	271	78.6
	Benzodiazepine	29	8.4
	Alcohol (all forms)	18	5.2
	Antibiotic	4	1.2
	Antidepressant	4	1.2
	Antipsychotic	4	1.2
	Paracetamol	4	1.2
	Other	11	3.2
Psychosocial stressor	Interpersonal	140	40.6
	Familial	66	19.1
	Marital	62	18
	Financial	17	4.9
	None	36	10.4
	Physical illness	5	1.4
	Secondary to alcohol use	5	1.4
	Death of family member	4	1.2
	Work related	4	1.2
	Other	6	1.7
Place of poisoning	Home	333	96.5
	Hostel	5	1.4
	Workplace	5	1.4
	College	1	0.3
	Bus	1	0.3
Route of exposure	Oral	341	98.8
	Inhalation	4	1.2
Intent	Intentional	331	95.9
	Accidental	14	4.1

Table 3 presents the prevalence and types of psychiatric disorders among patients admitted for poisoning. Overall, 42.6% (n = 147) of patients had a psychiatric diagnosis. Mood disorders were the most common, affecting 25.2% (n = 88), with primary depression accounting for 21.7% (n = 75). Other diagnoses included adjustment and stress-related disorders (3.8%, n = 13), personality disorders (3.8%, n = 13), psychotic disorders (2.9%, n = 10), and anxiety or somatoform disorders (2.6%, n = 9). Substance use disorders were identified in 2% (n = 7) of patients, while 1.4% (n = 5) had other issues, such as insomnia.

**Table 3:** Prevalence of psychiatric disorders in patients with poisoning at a tertiary care hospital in Chennai (N = 345)

Psychiatric diagnosis	Frequency	Percentage (%)
No psychiatric disorder	198	57.4
Mood disorders		
Depression (primary)	75	21.7
Depression with alcohol dependence syndrome (ADS)	3	0.9
Depression with personality disorder	3	0.9
Postpartum depression	1	0.3
Dysthymia	2	0.6
Bipolar affective disorder (BPAD)	2	0.6
Grief reaction	1	0.3
Pathological grief	1	0.3
Subtotal	88	25.2
Adjustment and stress disorders		
Adjustment disorder	12	3.5
Acute stress reaction	1	0.3
Subtotal	13	3.8
Personality disorders		
Personality disorder	9	2.6
Emotionally unstable personality disorder (EUPD)	3	0.9
Attention-deficit/hyperactivity disorder (ADHD) with personality disorder	1	0.3
Subtotal	13	3.8
Other neurotic and somatoform disorders		
Anxiety, not otherwise specified (NOS)	6	1.7
Generalized anxiety disorder	1	0.3
Obsessive compulsive disorder	1	0.3
Dissociative disorder	1	0.3
Somatoform disorder	1	0.3
Subtotal	10	2.6
Psychotic disorders		
Schizophrenia	6	1.7
Psychosis NOS	4	1.2
Subtotal	10	2.9
Substance use disorders		
ADS	5	1.4
Polysubstance abuse	2	0.6
Subtotal: substance use disorders	7	2
Other disorders		
Insomnia	5	1.4

Table 4 shows the differences in clinical characteristics between patients with and without psychiatric diagnoses. There was no statistically significant difference (p > 0.05) in the mean time from poisoning to hospital presentation between patients with psychiatric diagnoses (4.51 hours) and those without (4.8 hours). Similarly, the mean length of hospital stay did not differ significantly, with an average of 4.35 days for patients with psychiatric diagnoses and 3.77 days for those without.

**Table 4:** Comparison of clinical variables by psychiatric diagnosis status in patients with poisoning at a tertiary care hospital in Chennai (N = 345)

Variable	Psychiatric diagnosis	N	Mean	Standard deviation	Standard error mean	95% confidence interval (lower-upper)	p-value
								Time interval (hours)	Present	147	4.51	4.1	0.34	-1.53 to 0.95	0.643
Absent	198	4.8	6.77	0.48	-1.45 to 0.86										
						Hospital stay duration (days)	Present		147	4.35	4.33	0.36	-0.24 to 1.40	0.164	
Absent	198	3.77	3.4	0.24	-0.27 to 1.43										

Table 5 summarizes the association between psychiatric diagnostic status and sex. There was no statistically significant association between gender and the presence of a psychiatric diagnosis (p = 0.636). However, a higher proportion of females (68.7%, n = 101) than males (31.3%, n = 46) were diagnosed with psychiatric disorders.

**Table 5:** Sex distribution by psychiatric diagnosis status in patients with poisoning at a tertiary care hospital in Chennai (N = 345)

Psychiatric diagnosis	Female	Male	Total	p-value			
Present	101 (68.7%)	46 (31.3%)	147 (100.0%)	(χ² = 0.253, df = 1, p = 0.636)			
Absent	141 (71.2%)	57 (28.8%)	198 (100.0%)				
Total	242 (70.1%)	103 (29.9%)	345 (100.0%)				

Table 6 presents the distribution of age groups among patients with and without psychiatric diagnoses. A statistically significant difference in age group distribution was observed between the two groups (p = 0.002). Among patients with psychiatric diagnoses, 36.1% (n = 53) were aged 18-30 years, 29.9% (n = 44) were 31-45 years, and 20.4% (n = 30) were 46-60 years. In contrast, the majority of patients without psychiatric diagnoses belonged to the youngest age group (56.6%, n = 112).

**Table 6:** Age distribution by psychiatric diagnosis status

Psychiatric diagnosis	18-30 years	31-45 years	46-60 years	61-75 years	76-90 years	Total	p-value
Present	53 (36.1%)	44 (29.9%)	30 (20.4%)	16 (10.9%)	4 (2.7%)	147 (100.0%)	(χ² = 16.620, df = 4, p = 0.002)
Absent	112 (56.6%)	33 (16.7%)	31 (15.7%)	20 (10.1%)	2 (1.0%)	198 (100.0%)	
Total	165 (47.8%)	77 (22.3%)	61 (17.7%)	36 (10.4%)	6 (1.7%)	345 (100.0%)	

Table 7 presents the distribution of psychosocial stressors among patients with and without psychiatric diagnoses. A statistically significant association was observed between psychiatric diagnostic status and the distribution of documented psychosocial stressors (p = 0.041). Interpersonal stressors were more frequently reported among patients without psychiatric diagnoses, whereas family and marital stressors were comparably represented in both groups. Certain stressors, including alcohol-related issues and loss of a family member, were predominantly documented among patients with psychiatric diagnoses (Figure 3).

**Table 7:** Distribution of stressors by psychiatric diagnosis status *More than one stressor.

Stressor type	Psychiatric diagnosis present	Psychiatric diagnosis absent	Total	p-value			
Interpersonal	52 (35.4%)	88 (44.4%)	140 (40.6%)	(χ² = 24.378, df = 14, p = 0.041)			
Familial	33 (22.4%)	33 (16.7%)	66 (19.1%)				
Marital	27 (18.4%)	35 (17.7%)	62 (18.0%)				
No stressor	14 (9.5%)	22 (11.1%)	36 (10.4%)				
Financial	3 (2.0%)	14 (7.1%)	17 (4.9%)				
Death of family member	3 (2.0%)	1 (0.5%)	4 (1.2%)				
Physical illness	3 (2.0%)	2 (1.0%)	5 (1.4%)				
Secondary to alcohol use	5 (3.4%)	0 (0.0%)	5 (1.4%)				
Work related	2 (1.4%)	2 (1.0%)	4 (1.2%)				
Combined stressors*	4 (2.7%)	1 (0.5%)	5 (1.4%)				
Secondary to psychotic symptoms	1 (0.7%)	0 (0.0%)	1 (0.3%)				
Total	147 (100.0%)	198 (100.0%)	345 (100.0%)				

**Figure 1:**
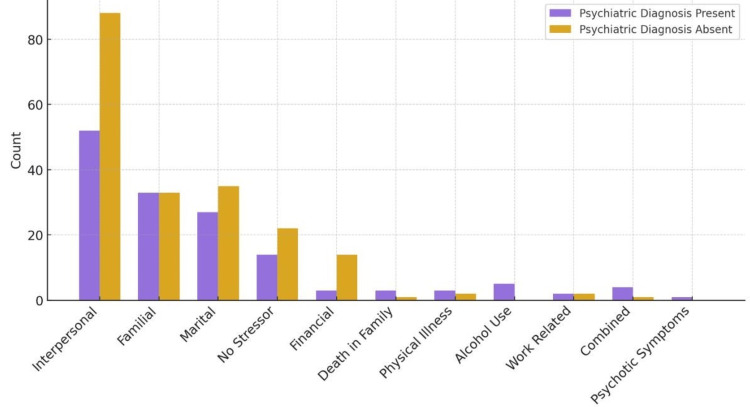
Graphical presentation of stressor distribution by psychiatric diagnosis status

Table 8 presents the correlations between psychiatric diagnostic status and selected clinical variables. A statistically significant negative correlation was observed between the presence of a psychiatric diagnosis and the time interval between poisoning attempts (r = -0.225, p < 0.05), indicating that individuals with a psychiatric diagnosis tended to have shorter intervals between repeated self-poisoning events. Additionally, psychiatric diagnosis showed a small but statistically significant positive correlation with type 2 diabetes mellitus (r = 0.124, p < 0.05).

**Table 8:** Correlation of psychiatric diagnosis with interval between attempts and medical comorbidities *Correlation is significant at the 0.05 level (2-tailed). **Correlation is significant at the 0.01 level (2-tailed).

Variable 1	Variable 2	Pearson correlation	Sig. (2-tailed)	N
Psychiatric diagnosis	Duration between attempts	-0.225**	0	345
Psychiatric diagnosis	Type 2 diabetes mellitus	0.124*	0.022	345
Psychiatric diagnosis	Hypothyroid	0.056	0.301	345
Psychiatric diagnosis	Anemia	-0.023	0.671	345
Type 2 diabetes mellitus	Hypothyroid	0.046	0.393	345
Type 2 diabetes mellitus	Anemia	0	0.995	345
Hypothyroid	Anemia	-0.055	0.309	345

## Discussion

Our study provides a comprehensive overview of the sociodemographic, clinical, and psychiatric characteristics of patients admitted with poisoning to a tertiary care hospital in Chennai. The findings are consistent with previous studies from South Asia [15-17] and highlight the multifactorial nature of poisoning, particularly among young individuals who are vulnerable to psychosocial stressors and societal pressures. Most patients were aged 18-30 years, and females constituted a substantial majority (70.1%, n = 242). Similar trends were reported by Kumbhar Ganesh et al., who observed the highest prevalence of poisoning in the 21-30-year age group, with females accounting for 60.14% of cases [15]. These patterns likely reflect psychosocial stressors, societal expectations, and gender-specific pressures among young women, rather than individual deficits in coping.

In our cohort, 95.9% (n = 331) of cases involved intentional poisoning, indicating that self-harm and suicidal behavior are the predominant drivers. This aligns with findings from Rahman et al. and Kafle et al., who reported that most poisoning cases were associated with suicidal intent, often related to marital and family stressors [16,17]. Similar stressors were identified in our study, with interpersonal, familial, and marital pressures being prominent. Women, particularly housewives, were disproportionately affected, suggesting that despite their central role in maintaining family well-being, their mental health needs may be overlooked.

Corrosive agents were the most frequently consumed substances in our study. This contrasts with other Indian studies, including those by Kumbhar Ganesh et al., Badiadka et al., and Naik et al., which reported higher rates of organophosphate and pesticide poisoning [15,18,19]. This difference may be explained by the predominance of housewives from semi-urban settings in our cohort, where corrosive cleaning agents are readily accessible. The use of prescription medications such as benzodiazepines and antidepressants may reflect urban residence and prior exposure through treatment of the patient or family members for mental health conditions.

Psychiatric morbidity was identified in 42.6% (n = 147) of patients, with depressive disorders being the most common, followed by adjustment disorders, personality disorders, and anxiety disorders. This distribution is comparable to findings by Naik et al., who reported high levels of impulsive suicidal behavior and psychosocial stressors among adolescents [19]. Although our study focused on adults, similar patterns emerged, suggesting that unresolved psychosocial stressors may persist into adulthood when mental health needs are inadequately addressed. The predominance of young housewives and depressive disorders underscores the need for strengthened psychosocial assessment and support systems rather than implying direct causation. As psychiatric diagnoses were based on retrospective documentation, under-recognition is possible, and causality cannot be inferred.

No statistically significant differences were observed in time to hospital presentation or length of hospital stay between patients with and without psychiatric diagnoses. However, psychiatric comorbidity was more frequently identified among females and individuals aged 18-30 years. These findings highlight the importance of routine psychiatric screening in younger patients and women presenting with poisoning, rather than suggesting differences in healthcare-seeking behavior or clinical severity.

Most poisoning events occurred at home and involved oral ingestion, consistent with reports by Kumbhar Ganesh et al., Rahman et al., and Chatterjee et al. [15,16,20]. This emphasizes the need for home-based safety interventions, improved mental health screening, and strategies to limit access to potentially harmful substances.

This study has several limitations. Its retrospective design relied on the accuracy and completeness of medical records, introducing potential reporting bias. Being a single-center study conducted in an urban tertiary care hospital limits generalizability. Variability in documentation of psychosocial stressors may have resulted in information bias. Mild poisoning cases not referred to tertiary care and patients declared dead on arrival were excluded, leading to possible selection bias. Psychiatric diagnoses were based on routine clinical assessments rather than standardized instruments, raising the possibility of misclassification, and the absence of follow-up data limited evaluation of recurrence and mortality.

Overall, our findings support existing regional and national literature and demonstrate that psychiatric morbidity is common among survivors of poisoning. The predominance of young, married women with interpersonal stressors, combined with ready access to prescription medications and corrosive substances, highlights the need for integrated psychiatric care, community-based mental health services, and gender-sensitive prevention strategies. While implementation may be constrained by infrastructure limitations and uneven distribution of mental health services, strengthening referral pathways and providing basic psychosocial training for non-psychiatric clinicians may represent practical initial steps toward improving early identification and intervention.

## Conclusions

Our findings suggest that, in this tertiary care center in Chennai, nearly half of deliberate poisoning cases occurred among young married women, predominantly within domestic settings. Psychiatric diagnoses were identified in more than two-fifths of patients. Given the high proportion of intentional poisoning and significant psychiatric comorbidity, integrating consultation-liaison psychiatric services into emergency departments and hospital settings may facilitate timely psychiatric assessment and mental health support as part of routine care for poisoning patients. In addition, restricting access to hazardous substances and increasing public awareness may help reduce poisoning incidence and improve both clinical and psychosocial outcomes in vulnerable populations. Public health initiatives should focus on improving mental health literacy, promoting early recognition of warning signs, and strengthening community-based support for at-risk groups.

## Appendices

**Table 9:** Results of normality tests for continuous variables

Variable	N	Shapiro-Wilk W	p-value
Time interval from poisoning to presentation (hours)	345	0.782	<0.001
				Hospital stay duration (days)	345	0.845	<0.001
Age (years)	345	0.964	<0.001
